# Suspect Guillain-Barré syndrome in a male rhesus macaque (*Macaca mulatta*)

**DOI:** 10.5194/pb-4-27-2017

**Published:** 2017-03-07

**Authors:** Tamara Becker, Florian Pieper, David Liebetanz, Martina Bleyer, Annette Schrod, Kerstin Maetz-Rensing, Stefan Treue

**Affiliations:** 1 German Primate Center, 37077 Göttingen, Germany; 2 Georg August University, University Hospital, 37075 Göttingen, Germany

## Abstract

Guillain-Barré syndrome (GBS) is a rare, mainly acute inflammatory polyneuropathy in humans. It is frequently post-infectious with auto antibodies being formed against myelin sheaths, resulting in a progressive and more-or-less severe paralysis of the motor neuron and cranial nerves. Mortality is low and 60 % of the patients recover completely from the disease after intensive treatment.

In animals, there are a few diseases that closely resemble GBS, but cases of GBS in monkeys seem to be scarce. In this case report, the clinical course of a progressive tetraplegia in a male rhesus macaque is described. Clinical, cerebrospinal fluid (CSF), electroneurography (ENG) and electromyography (EMG), and pathological findings revealed symptoms very similar to human GBS.

## 1 Introduction

Guillain-Barré syndrome (GBS), first described in 1916 by French neurologists Jean-Alexandre Barré and Georges Charles Guillain, is a mainly acute, incidentally chronic, inflammatory polyneuropathy affecting the myelin-protein sheathing and the axons of spinal nerve roots and peripheric nerves (motor neuron) in humans.

It is a frequently post-infectious, immune-mediated disease with mainly immunoglobulin G (IgG)auto-antibodies being formed against endogenous myelin and gangliosides.

There are numerous infectious agents, either viral, bacterial, or even parasitic, that can trigger the onset of GBS such as herpes viruses, i.e., varicella zoster virus (Creswell et al., 2010), Epstein–Barr virus (Kim et al., 2016) or cytomegalovirus (Steger et al., 2012); human immunodeficiency virus, HIV (Varshney et al., 2014); mycoplasma (Topcu et al., 2013); *Brucella melitensis* (Namiduru et al., 2003); *Campylobacter jejuni* (*C. jejuni*); *Toxoplasma gondii* (Pascual et al., 1984); or influenza vaccines (Martìn Arias et al., 2015). Preceding *C. jejuni* infection can be found in a high percentage of GBS patients. This association was initially reported in the early 1980s (Kaldor and Speed, 1984). Recent publications report that GBS is also associated with the Zika virus infection (Cao-Lormeau et al., 2016). In animals, GBS-like diseases have been reported following a rabies (Hemachudha et al., 1988) or swine influenza vaccination (Langmuir et al., 1984).

Myelin sheath antigens and *C. jejuni* antigens show an equal configuration, which can lead to a cross-reaction of the antibodies. It is assumed that at least some strains of *C. jejuni* express a carbohydrate epitope on their lipopolysaccharides that can also be found on peripheral nerves (Wirguin et al., 1994).

Why some patients develop GBS after an infectious disease while others do not is so far unknown.

There is an average of 1–2 cases yr${}^{-1}$ in 100 000 people worldwide (Fujimura, 2013). An age accumulation in humans at the ages of 20–30 and 50–70 can be seen, but people at every age can be affected (Sindern and Malin, 1996).

The main symptom of GBS is an ascending paralysis with a mild to severe progression up to total paralysis including the respiratory muscles, cranial nerves and, in the worst case, affecting the autonomic nervous system (heart frequency, circulatory system, temperature regulation and urination). While sensitivity is preserved, more than 60 % of the patients suffer from mild to severe myalgia (Ruts et al., 2010).

Although the disease can last for weeks to months, mortality in humans is low (about 5 %; Sindern and Malin, 1996). With adequate treatment, 60 % of the patients show a total recovery or remission. However, more-or-less mild symptoms remain, e.g., some weakness, muscle wasting, impaired walking or pain in some patients. The most severe, therapy-resistant cases can lead to high tetraplegia and persistent failure of cranial nerves (Tan and Chee, 1995).

GBS can mainly be diagnosed through clinical findings. An anamnesis often reveals previous infectious diseases, trauma or severe stress. Blood analysis can yield a high lymphocyte count, while there are no special findings in the CSF (cerebrospinal fluid) at the beginning of paralyses, whereas in the disease's progression total CSF protein rises due to the swelling of myelin sheaths resulting in an impaired CSF flow (Reiber, 1994, 2016). In most cases CSF cell count remains normal. In case of a suspected GBS the reduced muscular and nerve conduction can be monitored by means of electromyography (EMG) and/or electroneurography (ENG).

There is no causal therapy for GBS. Patients need intensive care according to the degree of severity. If needed, artificial respiration as well as a urinary catheter has to be provided, and prophylaxis of decubitus and thrombosis is required. The application of glucocorticoids to reduce the immune reaction and that of immunoglobulins as well as plasmapheresis for the reduction of autoimmune antibodies, complement and mediators of inflammation, can be helpful (Sindern and Malin, 1996; Yuki and Hartung, 2012).

## 2 GBS-like diseases in animals

Several GBS-like illnesses have been described in different animal species, either naturally occurring or experimentally induced. Coonhound paralysis (CHP), first described in 1954, resembles human GBS and can be found in coonhounds 1–2 weeks after a raccoon bite or scratch (Kingma and Catcott, 1954). CHP could also be experimentally reproduced through the injection of raccoon saliva into healthy coonhounds (Holmes et al., 1979). Idiopathic acute polyradiculoneuropathy is the most commonly recognized peripheral neuropathy in dogs that closely resembles the acute axonal or intermediate form of human GBS and has been observed following rabies vaccination (Hariharan et al., 2011; Cuddon, 1998; Collins, 1994). Rabbits, guinea pigs, mice and rats are used as widely accepted animal models for GBS. In these laboratory animal species, so-called experimental autoimmune neuritis can be induced by injecting peripheral nerve emulsion in complete Freund's adjuvant (Smith et al., 1979; Saida et al., 1981). Marek' s disease in chickens has formerly been discussed as another natural model for GBS as it leads to an autoimmune response to myelin and peripheral nerves; however, it is initiated by a viral infection in neuronal supporting cells (Hariharan et al., 2011).

In horses, cauda equina neuritis also clinically resembles GBS, and circulating antibodies to a myelin protein have been detected in affected animals (Kadlubowski and Ingram, 1981). Upper respiratory infections involving streptococci have been discussed as a cause (Martens et al., 1970).

Cases of GBS or GBS-like diseases in nonhuman primates seem to be rare. There is one single report of a chimpanzee (*Pan troglodytes*) that, after several months of treatment, made a total recovery from a GBS-like condition with albuminocytologic dissociation in CSF following a rabies revaccination and tooth extraction (Alford and Satterfield, 1995). Another case about an entellus langur (*Presbytis entellus*) with a four-week duration of clinical signs and a tenfold rise of CSF proteins during the course of the disease (Schultz, 1987) is known.

We here report about an eight-year-old rhesus macaque (*Macaca mulatta*) showing tetraplegia after previous campylobacteriosis.

## 3 Case Report

### 3.1 Anamnesis, Patient

The patient, Simon, was a male rhesus macaque. When the clinical signs appeared, he was eight years old and weighed 9 kg. He was born in China and imported to Europe via the Netherlands at the age of four. Since then, the monkey was kept at the Department of Cognitive Neuroscience at the German Primate Center. Simon was tested Herpes-B negative and group housed with three other B-virus negative males, with the present group existing since 18 months. Simon wore two chronic head implants, i.e., a titanium head post (∼ 3 years) and a recording chamber over a craniotomy (∼ 1 year) fixed to the bones of the skull. He was trained for his monkey chair, performing self-controlled neuroscientific experiments on a weekday basis. During the experimental sessions (∼ 1–2 h) a single parylene-C coated tungsten electrode was introduced into the upper cortical layers of the brain using a guide tube to just penetrate the dura under the craniotomy. This allowed the recording of single neuron's responses during the given experimental task. All procedures were done under locally sterile conditions.

Two weeks prior to the events reported here, the monkey had suffered from mild to moderate diarrhea with *C. jejuni* isolated from the feces. In a routine microbial sensitivity test the antibiotic Cefquinome was found highly efficient and a therapy with Cobactan^®^ (2 mg Cefquinome kg body weight${}^{-1}$) was applied for five consecutive days.

### 3.2 Clinical Course

### Day 1

Around noon, Simon was found in his cage not being able to move his hind limbs. Apart from that, his general condition seemed to be unimpaired.

A visual veterinary examination confirmed these findings, with the left leg being even more severely affected by sudden lameness than the right and the muscles of both hind limbs appearing atonic. An immediate clinical examination under Ketamine sedation (10 mg kg${}^{-1}$) revealed no special findings in particular and no injuries such as hematomas, wounds or fractures either on the legs or on other body parts. A blood sample was taken (serum, EDTA) for general routine blood works and Prednisolone (1.0 mg kg${}^{-1}$) as well as Meloxicam (0.2 mg kg${}^{-1}$) were injected. A blood analysis was performed on the same day and revealed no special findings.

### 3.3 Differential Diagnoses

At this point several differential diagnoses were discussed. First of all a spinal cord tumor was considered, such as a malignant lymphoma caused by the Epstein–Barr virus, as it had been found in another monkey in the same unit 20 months earlier. Any other tumor of either the spinal cord or vertebral column was also conceivable, as was any other type of compressive lesion, e.g., a herniated disk. There could have been a brain injury due to the insertion of an electrode during the experiment; however, the electrodes were never inserted near the motor cortex and there had been no recording in the last 48 h. Additionally, a spinal cord infarction could have taken place. Despite no visible external injury and without the caretakers having observed any accident or fight, a spinal cord trauma could have still happened. Acute muscle degeneration or neurological diseases, e.g., myasthenia gravis, and even highly improbable options like botulism or other neurotropic intoxications had to be taken into account as well. Since Simon was wearing chronic head implants, we also thought about a possible unrecognized infection of the brain or its surrounding tissues even if there had been no indication of a recording chamber infection or an infection of the skin margins around the head post prior to this clinical event.

**Table 1:** Analysis of two different CSF and serum samples (a and b) taken on day 2 and 7, respectively. Since cytology required fresh CSF while these samples were frozen at -80 ${}^{\circ }$C prior to analysis, cells could not be differentiated into lymphocytes, monocytes, granulocytes, and plasma cells and thus were labeled “other cells”. Reference value for rhesus monkeys for albumin is 29–228 mg L${}^{-1}$ and 0.5–4.3 for albumin ratio (Q Albumin). Total protein should not exceed 60–350 mg L${}^{-1}$ and cell count should be < 4 µL; reference value for IgG is 6–50 mg L${}^{-1}$ and 0.4–4.0 for IgG ratio (Q IgG) (Smith and Lackner, 1993). The counts that exceed the references are marked in bold.

	CSF		Serum × 10${}^{3}$		Q (CSF/Ser) × 10${}^{3}$	
	a	b	a	b	a	b
Albumin mg L${}^{-1}$	61.8	**295**	24.8	24.6	2.5	**12.0**
IgG mg L${}^{-1}$	10	45.2	8.7	8.4	1.1	**5.4**
IgA mg L${}^{-1}$	0.6	2.9	0.91	0.92	0.7	3.1
IgM mg L${}^{-1}$	< 0.14	0.54	0.38	0.4	< 0.4	1.4
Total protein mg L${}^{-1}$	145	**542**				
Cells µL	2	**29**				

### 3.4 Clinical Course

### Day 2

On day 2 Simon's condition seemed to be unchanged. With the help of his arms he pulled himself through the cage, apparently coping quite well with his situation and eating and drinking as usual.

We injected Dexamethasone (0.2 mg kg${}^{-1}$, i.m.) and Enrofloxacin (5 mg kg${}^{-1}$, s.c.) and gave him Vitamin B complex supplements (2.5 mL p.o.).

The monkey was then sent under sedation to Hannover's veterinary school for an X-Ray and a CT scan. A clinical examination as well as a spinal tap and an immediate CSF analysis were performed by veterinary neurologists of the veterinary school's small animal hospital. All these efforts submitted no special findings as the suspicion of a spinal cord infarction was discussed. The specialists suggested waiting for about two weeks and recommended medication for bladder constriction (Bethanechol chloride, Myocholine^®^) and sphincter relaxation (Phenoxybenzamine hydrochloride, Dibenzyran^®^) to support urinary bladder function. The administration and continuation of glucocorticoids, both for immune suppression and to control local inflammation, antibiotics and supplementation with Vitamin B were recommended.

### Day 3 and 4

The third day found Simon in an unchanged condition. He showed spontaneous defecation and surprisingly scratched his right leg after an injection, indicating at least a certain degree of sensitivity.

Medication was administered as recommended: Myocholine^®^ (10 mg day${}^{-1}$, p.o.), Dibenzyran^®^ (10 mg day${}^{-1}$, p.o.), Enrofloxacin, Dexamethasone and Vitamin B supplements.

His general condition was still good and, besides the reaction to the needle puncture, he showed no signs of pain.

The next day was the same, although the caretakers reported polyphagia, polydipsia and polyuria, which did not come unexpectedly due to the glucocorticoid therapy.

### Day 5

Since the monkey got quite dirty from dragging himself around in the cage and through his feces, we washed and dried him under sedation and added soft and dry bedding to his cage. Furthermore, we tried some physiotherapy by massaging and passively moving his legs for a while.

Medication, as above, was continued.

### Day 6

On the morning of day 6 the caretakers found Simon only being able to handle large pieces of food. Both of his hands were flaccidly paralyzed, his fingers were bent and both fists were atonic. He reclined a lot and had trouble sitting up. His arms seemed to be weaker than the days before.

After a second consultation of the veterinary neurologists, GBS was suspected.

### Day 7

On day 7, Simon's condition worsened. He was in lateral recumbency, sometimes resting on one elbow and rolling over to change his position. He was still able to eat, drink and defecate and showed no signs of pain.

Another CSF and blood sample was taken and all CSF and blood samples were sent to the neurochemistry lab of the Göttingen university hospital.

**Figure 1:**
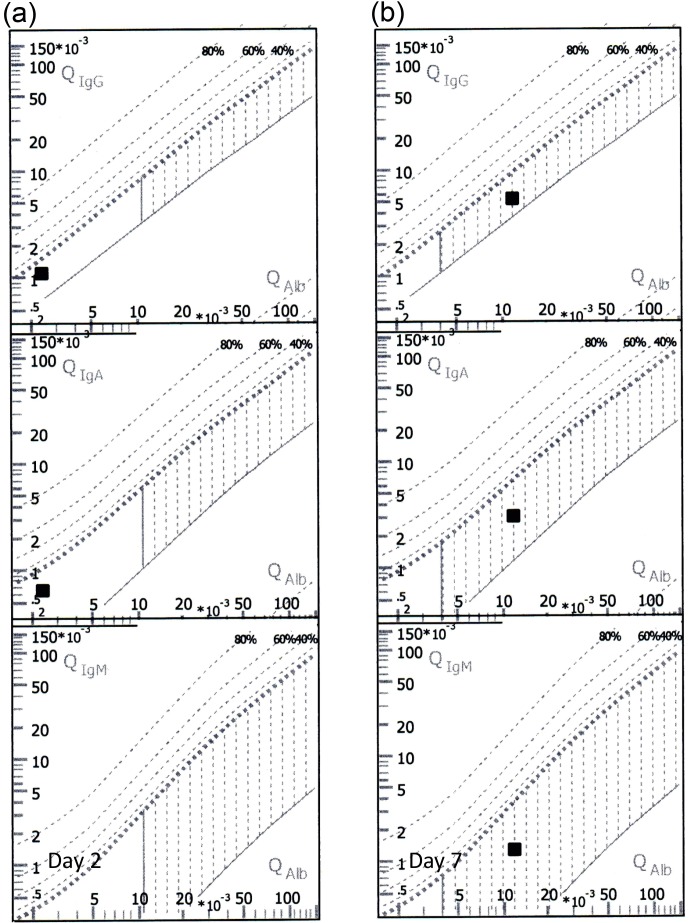
Reibergrams for IgG, IgA, IgM, and albumin ratios in CSF and serum samples **(a)** and **(b)** (see Table 1) taken on day 2 (left, CSF/serum sample **a**) and day 7 (right, CSF/serum sample **b**), respectively. The position of the black square dot within the vertically hatched areas of the right reibergram (day 7) indicates a simple progressive disturbance of the blood-brain barrier, i.e., a reduced CSF flow, in comparison to day 2 (square dots are displayed within the blank normal area or below the limit). It also indicates that there is no intrathecal synthesis of antibodies, which would result in the black square dot being displayed within the percentage lines above the dotted line (Reiber, 2016).

As the blood counts revealed no special findings, there was a rise in total protein, albumin, IgG and cells in the second CSF sample in comparison to the first puncture (Table 1). Reibergrams displaying IgG, IgA, IgM and albumin ratios are shown in Fig. 1.

Oligoclonal IgG bands were identical in CSF and serum, indicating a systemic inflammation without intrathecal IgG synthesis.

We arranged electroneurography and electromyography measurements for the late afternoon of the following day.

### Day 8

On day 8, Simon's condition was unchanged, with the monkey being still in a lateral position and unable to use his arms and hands properly.

ENG and EMG, which could both be performed in the monkey unit using portable devices, revealed signs resembling those of advanced GBS in humans: ENG of both legs and arms indicated a highly reduced nerve conduction speed and a reduction of amplitudes as a sign of demyelination up to the nerve roots. EMG showed fibrillations and positive sharp waves in almost every tested peripheric muscle due to a serious axonal impairment, which can be seen secondarily to the demyelination in severe cases of GBS. Some back muscles, though, still showed normal potentials.

**Figure 2:**
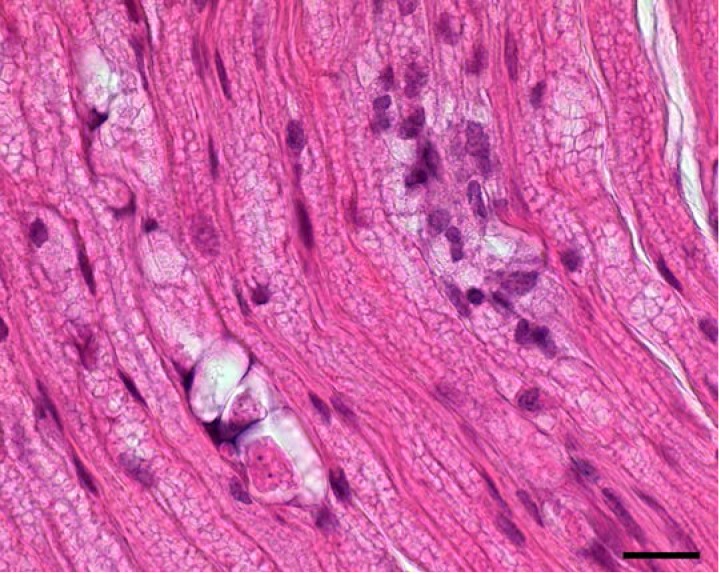
Mild polyneuritis, rhesus macaque (*Macaca mulatta*). Sciatic nerve with moderate vacuolar degeneration and focal lymphocytic infiltration. Scale bar: 20 µm.

Given these grave findings and the bad prognosis for a fast recovery as well as the impracticality of long-lasting intensive care for an adult male rhesus macaque, especially with regard to animal welfare, we decided to euthanize the monkey.

### Day 9

Simon was euthanized by intravenous injection of a barbiturate (Pentobarbital, 80 mg kg${}^{-1}$) under deep sedation (Ketamine, 5 mg kg${}^{-1}$ and Xylazine, 1 mg kg${}^{-1}$) and sent to DPZ's (German Primate Center) pathology department where an immediate autopsy was performed.

## 4 Postmortem

Macroscopic pathological findings consisted of a mild right heart dilatation and focal fibrosis of the myocardium of both ventricles. The right kidney showed moderate chronic interstitial nephritis and scarred contractions of the cortex. The left kidney was considerably larger with a remarkable striation of the cortex and suspect moderate diffuse interstitial nephritis. Lymph nodes in the pelvic cavity were mildly hyperplastic and there was mild chronic gastritis in the stomach's fundus region. Nervous tissue of both the pelvic and shoulder belt regions were macroscopically unnoticeable and were completely dissected for further histological examination. The central nervous system and all other organs appeared normal.

## 5 Histological findings

There were main findings in the nervous system. The sciatic nerve showed moderate vacuolar degeneration of myelin sheaths, segmental demyelination, moderate perineuritis with infiltration of lymphohistiocytic inflammatory cells and eosinophilic granulocytes in its passing region (Fig. 2).

Distal nerves, though, revealed no special findings. The macroscopic alterations in the kidneys, stomach and lymph nodes could be histologically verified. Additional findings related to mild chronic active enteritis, mild purulent tonsillitis with activation of germinal centers and mild reactive hepatitis were also revealed.

Bacteriological and parasitological findings remained unsuspicious.

## 6 Discussion

On the basis of clinical and pathomorphologic findings, a diagnosis of Guillain-Barré syndrome was made in the present case. GBS is a rare disease in humans and similar conditions have been described in no more than two monkeys of different species. The clinical course of this patient's paralysis, unsuspicious X-ray and CT scan, CSF findings, as well as ENG and EMG measurements closely resembled that of the human GBS. Furthermore, both postmortem and histological findings supported the clinical diagnosis. The mild inflammatory alterations found in the digestive tract, liver and heart could have been remnants of the anamnestically reported *Campylobacter jejuni* infection two weeks prior to the onset of paralysis. Several differential diagnoses, such as tumors, other compressive lesions or brain injuries could be ruled out by imaging techniques and pathological examinations.

Although the definition of GBS has been related to humans, several GBS-like diseases in animals exist. Given the fact that the nervous systems of nonhuman primates and humans are largely similar, the authors claim that this might be the first reported case of GBS in a rhesus monkey.

## 7 Data availability

The original data on serum and CSF analyses as well as on the postmortem and histological examinations can be provided upon request.
